# The Mechanisms Driving Vegetation Changes in Riparian and Typical Floodplains Under Cascade Hydropower Development in the Middle Reach of Hanjiang River

**DOI:** 10.3390/plants15030347

**Published:** 2026-01-23

**Authors:** Yiwen Liu, Xiaorong Lu, Zhiyuan Liu, Xuelei Wang, Qing Yang

**Affiliations:** 1 Key Laboratory for Environment and Disaster Monitoring and Evaluation of Hubei, Innovation Academy for Precision Measurement Science and Technology, Chinese Academy of Sciences, Wuhan 430077, China; liuyiwen@apm.ac.cn (Y.L.); liuzhiyuan@apm.ac.cn (Z.L.); 2 University of Chinese Academy of Sciences, Beijing 100049, China; 3 Advanced Interdisciplinary Institute of Environment and Ecology, Guangdong Provincial Key Laboratory of Wastewater Information Analysis and Early Warning, Beijing Normal University, Zhuhai 519087, China; yangqing14@mails.ucas.edu.cn

**Keywords:** riparian, floodplain vegetation, cascade hydropower development, driving mechanism

## Abstract

Vegetation within riparian and floodplain undergoes significant alterations driven by climatic factors and human interventions, particularly influenced by cascade hydropower development. This study investigated the dynamics of the Normalized Difference Vegetation Index (NDVI) in riparian and representative floodplains vegetation under cascade hydropower development in the middle reach of Hanjiang River by using Landsat imagery and hydroclimatic station data. The vegetation NDVI of the riparian increased significantly (*p* < 0.01) during the growing season, and the vegetation NDVI of the riparian and typical floodplains also increased after the hydropower developments. In terms of the key driving factors, the increased annual water level may explain the reduction in most floodplains. Increasing temperature, especially in March, can promote vegetation growth of riparian and typical floodplains. The development of cascade hydropower may result in different contributions of climate and hydrology to vegetation at different periods, and it was found that the climate is the major contributor to the changes in the vegetation NDVI after the construction of the dam. This research will help clarify the impact of cascade hydropower development on vegetation in riparian and floodplain ecosystems. It also provides a scientific basis for vegetation protection and environmental restoration in the basin.

## 1. Introduction

As the transition between aquatic and terrestrial ecosystems, the riparian ecosystem can promote the flow of species, energy, and nutrients [1,2,3,4]. Floodplains, widely distributed in riparian ecosystems [5], promote the hydrological cycle and have significant eco-hydrological value [6]. Vegetation as an important component of the riparian and floodplain can maintain the biodiversity, structure, and function of the floodplain ecosystem [7]. The Normalized Difference Vegetation Index (NDVI) is widely recognized as an indicator used to evaluate vegetation growth and it can effectively represent vegetation vigor and its spatial distribution features [8,9]. The NDVI has been widely applied to examine how hydroclimatic factors influence vegetation in riparian and floodplain ecosystems [10,11]. Existing studies have shown that riparian and floodplain vegetation show strong responsiveness to variations in hydrological and climatic conditions [12]. In recent years, influenced by hydrology and the climate, the vegetation in riparian and floodplains has also undergone significant changes [12,13]. Moreover, the growing season was selected to better capture vegetation growth characteristics, and empirical methods have been widely applied to determine the growing season, making it well suited for long-term historical analyses of the NDVI [9,10].

Cascade hydropower development refers to a group of hydropower projects in a stepped manner within the same basin, which can systematically utilize water resources [14,15]. The development of cascade hydropower profoundly affects the ecological hydrological processes of the region, and alters the regional climate to a certain extent, thereby exerting varying degrees of influence on the vegetation of the riparian and floodplains [16]. Several studies have examined how cascade hydropower development influences riparian vegetation [17,18]. For example, Liu et al. [17] found that cascade hydropower development can promote the growth of riparian vegetation in river basins of the Tibetan Plateau. The floodplains widely distributed in the riparian and the floodplains’ vegetation are vulnerable to the influence of hydrological and climatic conditions, especially those in the mid-channel and point bars. Several studies have further investigated how hydropower development affects vegetation in floodplains [19,20]. For example, Zeng et al. [20] found that the NDVI of floodplain vegetation in Poyang Lake increased after the development of the Three Gorges Dam. However, most of these investigations concentrated on the effects of single hydropower projects on floodplain vegetation, whereas research addressing the influence of cascade hydropower systems remains limited. Moreover, cascade hydropower development can trap sediment and control regional hydrological processes, which may lead to the change in the area and shape of point and mid-channel bars constantly [21,22]. The change in the area and shape of point and mid-channel bars also affects the distribution and growth of vegetation [23].

As the longest tributary of the Yangtze River Basin, the Hanjiang River serves as a crucial water source region for the Middle Route of the South-to-North Water Diversion Project and holds significant value in ecological and economic terms [24,25,26]. The extensive cascade hydropower development is distributed in the middle reach of Hanjiang River [24,25]. This area widely distributes floodplains, especially point and mid-channel bars [24,27]. At present, several researchers have investigated how cascade hydropower projects in the Hanjiang River Basin affect vegetation throughout the basin [27,28,29]. For example, Zhang et al. [27] found that the total mid-channel bar area between Xiangyang and Huangzhuang exhibited a strong negative relationship with runoff recorded at Huangzhuang hydrologic station. However, this study focused solely on the effects of cascade hydropower development on the mid-channel bar area, without examining its influence on the vegetation of point bars. Considering that basin vegetation is highly sensitive to climatic factors and human interventions, particularly the cascade hydropower development, in order to clarify how the cascade hydropower development affects the vegetation in the middle reach of Hanjiang River, it is necessary to investigate how hydrology and the climate impact the riparian and typical floodplain vegetation under cascade hydropower development.

Using Landsat data from 1990 to 2023, the research examines the spatiotemporal changes in the floodplain Normalized Difference Vegetation Index (NDVI) before and after cascade hydropower development in the middle reach of Hanjiang River. Additionally, based on monthly records from hydrological and meteorological stations between 1990 and 2023, this study examines the factors controlling dynamic vegetation change. This study aims to elucidate how cascade hydropower development affects vegetation in riparian and floodplain ecosystems, while offering a scientific foundation for vegetation conservation and restoration in the basin.

## 2. Results

### 2.1. Spatiotemporal Variations in NDVI in the Growing Season of Riparian Vegetation

**Figure 1:**
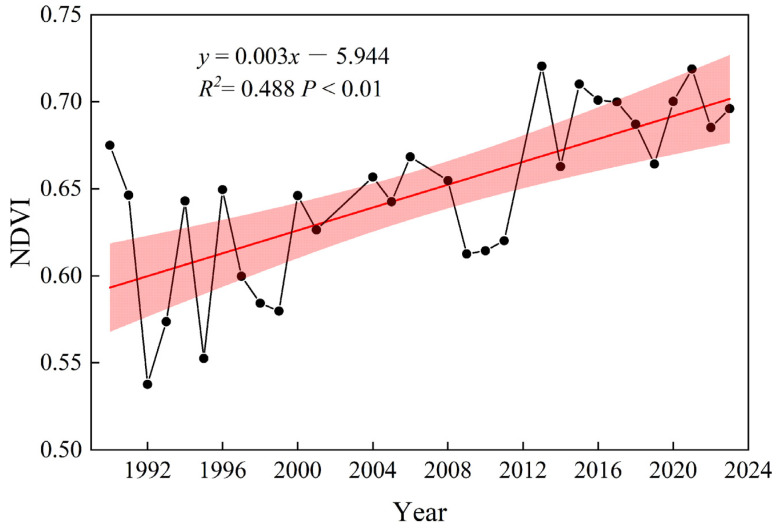
The trend of changes in NDVI of riparian vegetation during growing season in the middle reach of Hanjiang River from 1990 to 2023 (the shaded area represents the 95% confidence interval).

From 1990 to 2023, the NDVI of the riparian vegetation in the middle reach of Hanjiang River increased significantly (0.003/year, *p* < 0.01) during the growing season, indicating that vegetation growth improved and greening enhanced in the region (Figure 1). To assess how cascade hydropower development affects vegetation, we analyzed the influence of different hydropower stations on the vegetation NDVI during the growing season before and after dam construction and elevation. The average growing season NDVI increased after the completion of the Wangfuzhou dam (WFZ) in 2003, the first elevation increase in the Danjiangkou dam (DJK) in 2005, the completion of the Cuijiaying dam (CJY) in 2010, and the second elevation increase in the Danjiangkou dam (DJK) in 2013 (Table 1).

**Figure 2:**
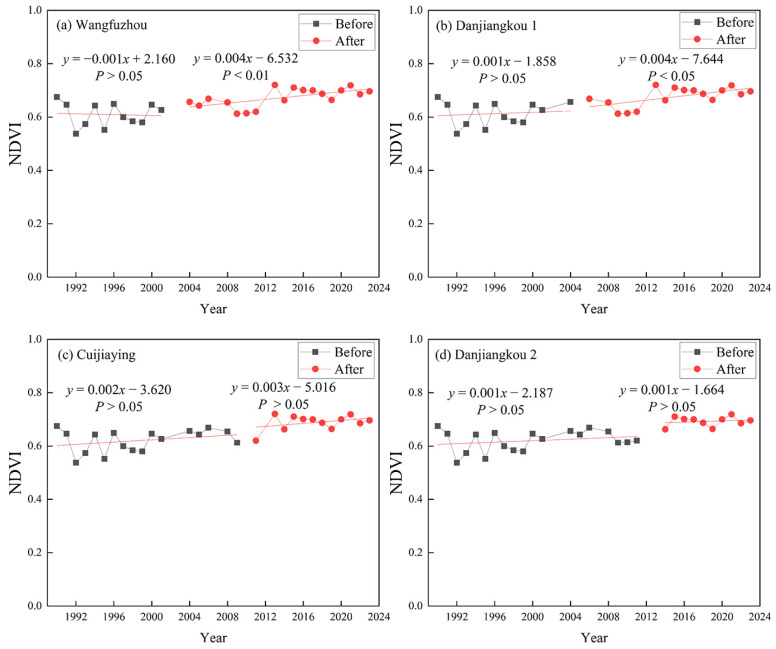
The trend of changes in growing season NDVI of riparian vegetation before and after the cascade hydropower development (Wangfuzhou was constructed and completed in 2003, Danjiangkou was first elevated in 2005, Cuijiaying was constructed and completed in 2010, and Danjiangkou was second elevated in 2013, *P* means significance).

Before the construction of the WFZ, the NDVI showed an insignificant decreasing trend during the growing season (0.001/year). However, after it, the NDVI increased significantly (0.004/year, *p* < 0.01) during the growing season (Figure 2a). Before the first elevation increase in the DJK, the NDVI increased insignificantly (0.001/year), while after it, the NDVI increased significantly (0.004/year, *p* < 0.05) (Figure 2b). Before and after the construction of the CJY, the NDVI showed an insignificant increasing trend during the growing season. However, after the construction of the CJY, the rising rate of the NDVI slightly increased (Figure 2c). The second elevation increase in the DJK had little impact on the growing season NDVI variation in the riparian vegetation (Figure 2d).

**Figure 3:**
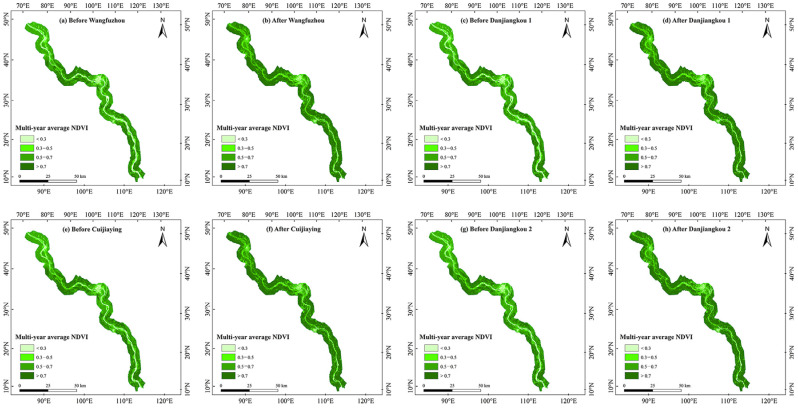
Spatial distribution of average growing season NDVI of riparian vegetation in the middle reach of Hanjiang River before and after cascade hydropower development.

**Figure 4:**
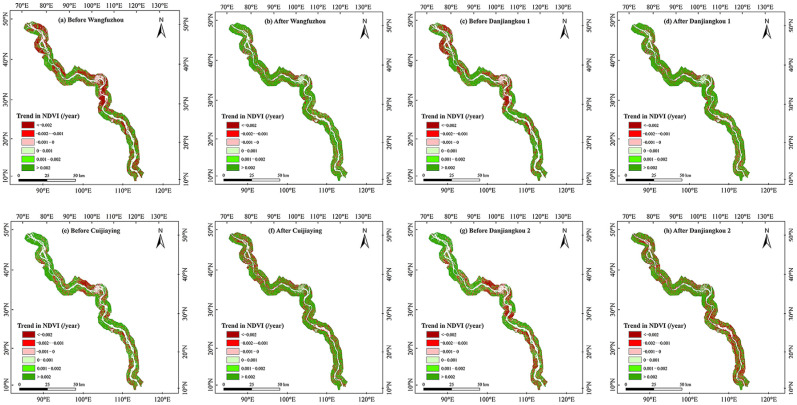
The spatial variation rates in growing season NDVI of riparian vegetation in the middle reach of Hanjiang River before and after the cascade hydropower development.

Spatially, we found that after the construction of the WFZ and CJY, and after the first and second elevation increases in the DJK, the average growing season NDVI of the riparian vegetation in this basin all showed an increasing trend (Figure 3). Regarding the trend of variation in the growing season NDVI of riparian vegetation, after the construction of the WFZ, the NDVI increased most significantly in the middle of the study area (Figure 4a,b). After the first elevation increase in the DJK, the majority of riparian vegetation showed an increasing NDVI trend in the growing season, while it increased most significantly in middle of this region (Figure 4c,d). After the construction of the CJY, the pixels of increased growing season NDVI of riparian vegetation were mainly appeared at the middle and south parts of the middle reach of Hanjiang River. The areas where the NDVI showed a decreasing trend were mainly distributed in the north of this region after the construction of the CJY (Figure 4e,f). After the second elevation of the DJK, the pixels of increased vegetation NDVI of riparian decreased, while the decreased NDVI mainly appeared in the south of the study region (Figure 4g,h).

For better understanding the vegetation changes in the riparian, we explored the correlation between the growing season NDVI of riparian vegetation and the climate and hydrology in this region. The results indicate that the growing season NDVI of riparian vegetation shows no significant correlations with annual or monthly precipitation. However, this study found that the NDVI showed a positive correlation with temperature, except in February and December, while the NDVI showed significant positive correlations with the temperatures in annual (*p* < 0.05) and March (*p* < 0.01) (Table 2). One possible reason for this result is that the initial growth of vegetation requires heat accumulation [30]. The increasing temperature in March can promote the heat accumulation of vegetation and promote its growth. On the other hand, it might be due to the cascade hydropower development that the regional climate has been changed, thereby promoting the growth of vegetation by influencing the regional climate. Insignificant relationships appeared in the growing season NDVI of riparian vegetation and monthly hydrological variables, which suggested that the effects of the water level on riparian vegetation under cascade hydropower development could not be significantly statistically detected in this study.

### 2.2. The Variations in Area of Typical Floodplains (Point and Mid-Channel Bars) and Their Influencing Factors

**Figure 5:**
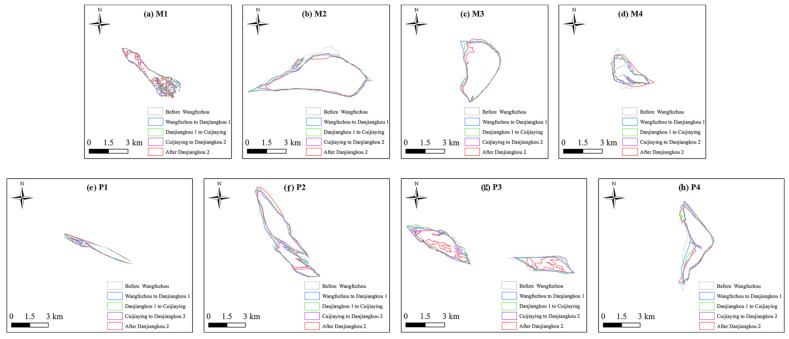
Morphological changes in different typical mid-channel and point bars (M means mid-channel bar and P means point bar, e.g., M1 means mid-channel bar 1, the same as following).

The shape and area of M1 showed no significant changes after the construction of the WFZ and CJY, but were greatly affected by the two elevation increases in the DJK (Figure 5a). After the first elevation increase in the DJK, the degree of M1 fragmentation increased, and the erosion in the southwest slightly increased. After the second elevation increase in the DJK, the fragmentation of M1 intensified, with an increase in erosion. The shape and area of M2 showed no significant changes after the cascade hydropower development. After the construction of the WFZ, the erosion was slight in the northern part of M2 (Figure 5b). The shape and area of M3 showed no significant changes after the cascade hydropower development. After the second elevation increase in the DJK, the erosion intensified in the northwestern part of M3, but the overall shape and area did not change significantly in M3 (Figure 5c). The shape and area of M4 showed no significant changes after the first elevation increase in the DJK and the construction of the CJY. After the construction of the WFZ, there was slight erosion in the northern part, the sedimentation appeared in the middle parts of M4, and the length from north to south decreased (Figure 5d). After the second elevation increase in the DJK, the area of M4 showed an increase.

The area and shape of P1 showed no significant changes after the construction of the CJY and the second elevation increase in the DJK. After the construction of the WFZ, the erosion of P1 increased and its area decreased (Figure 5e). After the first elevation increase in the DJK, a slight sedimentation appeared in P1 and the area of P1 was increased. The overall area and shape of P2 showed no significant changes after the construction of the WFZ and the two elevation increases in the DJK. After the construction of the CJY, the erosion of P2 increased, the overall area became smaller, and the length from east and west decreased (Figure 5f). The area and shape of P3 showed no significant variations after the construction of the WFZ and CJY and the first elevation increase in the DJK. After the second elevation increase in the DJK, the erosion of P3 intensified, its area decreased, and the level of fragmentation increased (Figure 5g). With the continuous intensification of cascade hydropower development, the overall area of P4 showed a decreasing trend, and especially the western side of P4 is gradually being eroded (Figure 5h).

**Figure 6:**
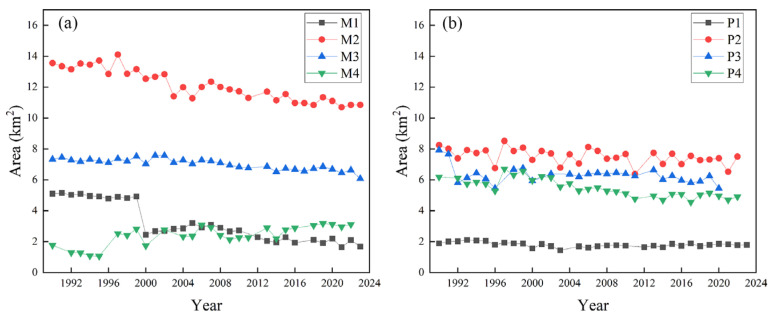
Changes in areas of mid-channel bars (**a**) and point bars (**b**) from 1990 to 2023.

This study further analyzed the trends in area changes in typical point and mid-channel bars in different regions of the middle reach of Hanjiang River. As is shown in Figure 6, except for M4, the mid-channel bars in different regions showed downward areas from 1990 to 2023, while the areas of most point bars were relatively changed inapparently. In particular, we found that compared with the smaller M1 and M4, the area of the larger M2 and M3 changed less (Figure 6). The reason for this might be that the larger mid-channel bars were more likely to be occupied and transformed by humans, thus receiving more protection and enhancing their stability [31]. In addition, the Hubei Changshou Island National Wetland Park was established along the northern riverbank of M2. Since the north riverbank reinforcement was completed in December 2017, the edge of M2 has been effectively protected and reinforced. Compared with other mid-channel bars, its morphological structure is more stable, so its area and shape have changed insignificantly.

For further clarifying the effects of the hydroclimate on typical floodplains, we analyzed the correlations between the area of typical floodplains (point and mid-channel bars) and annual precipitation and water level (Table 3, Table 4 and Table 5). The study found that the areas of point and mid-channel bars (except P4) showed negatively correlated with annual water level (Table 4 and Table 5). Among these, the negative correlations between the areas of M2, M3, M4, P1, and P2 and the annual water level were statistically significant (*p* < 0.05). That is, the higher the annual water level is, the smaller the area in the point and mid-channel bars is. A possible explanation for this is that a rising water level may inundate these point and mid-channel bars, thereby reducing their area [22]. The areas of the point and mid-channel bars showed insignificant correlations with the annual precipitation. The possible reason for this is that the increase in precipitation may indirectly affect the areas of point and mid-channel bars by influencing the water level, but the impact is not as significant as that of the water level.

### 2.3. Changes in Growing Season NDVI of Typical Floodplain Vegetation and Their Driving Mechanisms

**Figure 7:**
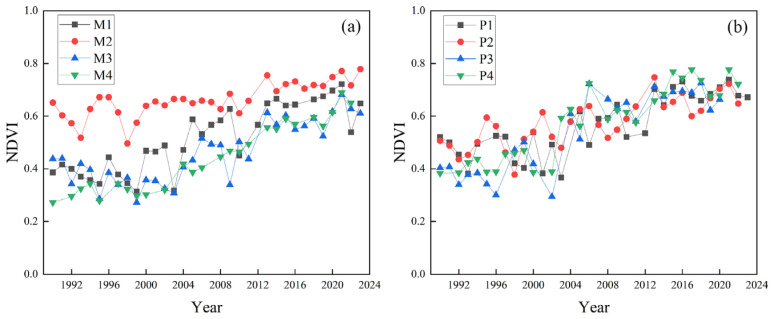
The changes in NDVI of typical mid-channel bars (**a**) and point bars (**b**) vegetation during growing season in the middle reach of Hanjiang River in 1990–2023.

Based on the annual boundaries of typical floodplains, this study analyzed the changes in the NDVI of typical floodplain vegetation during the growing season from 1990 to 2023. The results indicate that, following the effect of cascade hydropower development of the middle reach of Hanjiang River, the NDVI of typical point and mid-channel bars vegetation in different sections have all shown an upward trend from 1990 to 2023 (Figure 7). To analyze the influence of different hydropower stations on typical floodplains, we explored the variations in the average growing season NDVI of floodplain vegetation before and after the construction or elevation of hydropower stations. The research found that after the construction of the WFZ, the first elevation of the DJK, the construction of the CJY, and the second elevation of the DJK, the average NDVI of typical point and mid-channel bars vegetation all increased to varying degrees (Table 6), indicating that the growth of typical point and mid-channel bars vegetation in the region has improved and the degree of vegetation greening has increased.

Considering that the NDVI of riparian vegetation shows a weak correlation with water level (Table 2), we explored the influence of climate (temperature and precipitation) on the NDVI of typical point and mid-channel bars vegetation in the riparian during the growing season. No significant relationship was observed between the NDVI of mid-channel bars and annual precipitation, clarifying that the annual precipitation might not be the main cause affecting the NDVI of typical mid-channel bars vegetation during the growing season. The vegetation NDVI in the typical mid-channel bar exhibited a remarkably positive relationship (*p* < 0.01) with annual temperature, except at M1, suggesting that higher temperatures may enhance vegetation growth during the growing season. At different months, the correlation between the growing season NDVI of typical mid-channel bars vegetation and the precipitation was insignificant (Table 7). The NDVI (M1, M2, M3, and M4) of typical mid-channel bars vegetation showed significant positive correlation with the temperature in March. This phenomenon might be because the increasing temperature in March may be conducive to heat accumulation [30], thereby promoting the growth of vegetation. The vegetation NDVI at M2 during the growing season was significantly and positively correlated with temperatures from May to June (*p* < 0.05; Table 7), suggesting that higher temperatures during this period may enhance photosynthetic activity and consequently increase NDVI values. The vegetation NDVI at M2 and M3 showed a remarkably positive correlation with temperature from August to September, suggesting that warmer conditions during this period may stimulate vegetation growth and slow down vegetation senescence [32] and promote the increase in the vegetation NDVI of M2 and M3.

No significant relationship was found between annual precipitation and the vegetation NDVI of the typical point bar during the growing season (Table 8), indicating that the annual precipitation may not be the main cause affecting the NDVI of typical point bars vegetation during the growing season. The NDVI of typical point bars vegetation (except P1) exhibited significant (*p* < 0.01) positive correlations with the annual temperature, suggesting that the increasing annual temperature can promote vegetation growth in P2, P3, and P4, thereby increasing the NDVI. At different months, the study found that the vegetation NDVI of P1 showed a significantly negative relationship with precipitation in February (Table 8), indicating that the increasing precipitation in February may inhibit the growing season NDVI of vegetation increasing in this area. The reason might be that the initial growth of vegetation requires heat accumulation [30]. The increase in precipitation in February may cause a drop in temperature, resulting in insufficient heat accumulation and thereby inhibiting vegetation growth [33]. The vegetation NDVI of P1 and P4 showed a significant (*p* < 0.05) positive correlation with the temperature in March. This might be because the increase in temperature in March was conducive to heat accumulation [30], thereby promoting the growth of vegetation.

The vegetation NDVI of P2 during the growing season was significantly (*p* < 0.05) positively correlated with the temperature from March to June (except for April) (Table 8), indicating that the increase in temperature from March to June can promote vegetation growth in P2. The vegetation NDVI at P2 showed a significant positive correlation with August’s temperature, suggesting that warmer conditions in August could promote vegetation growth in P2. This might be that August is the month when vegetation grows most vigorously in the middle reach of Hanjiang River [34]. The increase in temperature in August can promote the activity of photosynthetic enzymes in vegetation and promote photosynthesis, thereby promoting the growth of vegetation [35]. The NDVI of vegetation at P3 exhibited a significant positive relationship with temperature during March–May, indicating that the increased temperature in spring (March to May) can increase the vegetation NDVI of the growing season in P3. The reason for this might be that the warmer temperature in spring will generally extend the growing season, therefore promoting the growth of plants [36]. The vegetation NDVI of P3 showed a significantly negative relationship with precipitation and a positive correlation with August’s temperature, indicating that excessive rainfall limited NDVI growth, while warmer conditions enhanced vegetation development. The possible reason may be that the increasing precipitation in August leads to a drop in temperature and the excessive precipitation may cause the water level to rise rapidly, flooding the vegetation and thus inhibiting vegetation growth [37]. August is the season when vegetation grows most vigorously in the middle reach of Hanjiang River [34]. The increase in temperature in August can promote the photosynthesis of vegetation [35], thereby promoting vegetation growth in P3.

### 2.4. The Influence of Hydroclimate on Vegetation NDVI Under the Cascade Hydropower Development

**Figure 8:**
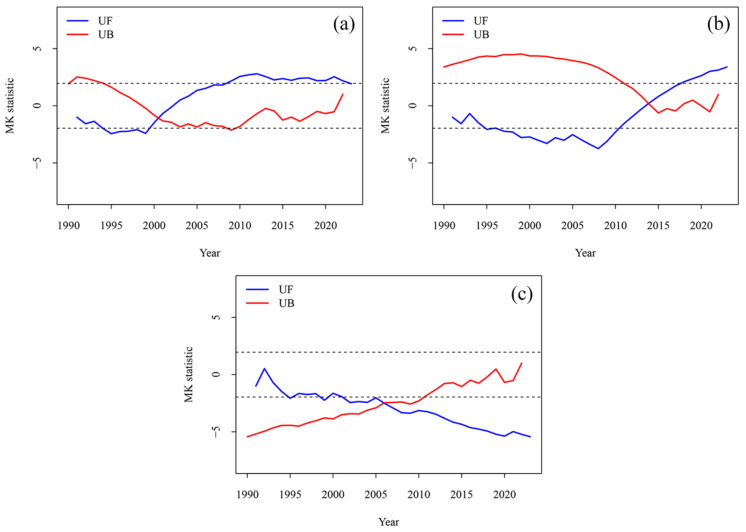
Abrupt change analysis for water levels (1990–2023). Hydrology stations: Huangjiagang (**a**), Xiangyang (**b**), and Huangzhuang (**c**). The horizontal and black lines represent the 95% confidence level.

Considering the impact of hydropower stations on hydrological regulation, we also conducted an abrupt change analysis of water levels (1990–2023). The intersection points within the ordinate values of ±1.96 (the 95% confidence level) were selected as significant abrupt changes (Figure 8). The study found that the abrupt change in the water level at Huangjiagang occurred in 2001 (Figure 8a), while it occurred in 2014 at Xiangyang (Figure 8b). The abrupt change at Huangzhuang was not significant (Figure 8c). Based on the construction time of the dams, it can be inferred that the abrupt change in the water level at Xiangyang was likely influenced by the combined effects of the Wangfuzhou dam, the heightenings of the Danjiangkou dam, and the Cuijiaying dam, leading to a certain degree of increase in regional water levels.

We employed segmented regression to further examine the relative importance of hydroclimate factors on the NDVI of riparian vegetation before and after the cascade hydropower development (Table 9). The study found that the relative contributions of climate and water level to the NDVI of riparian vegetation during the growing season changed to varying degrees before and after the operation of the hydropower stations. Before the operation of the Wangfuzhou dam (>75%) and before the first heightening of the Danjiangkou dam (>65%), the water level had a higher contribution to the variation in the NDVI of riparian vegetation during the growing season compared to climate. After their operations, however, the influence of the climate (precipitation +temperature) (>95%) became greater than that of the water level (Table 9). We inferred that this may be due to the operation of the Wangfuzhou dam and the first heightening of the Danjiangkou dam altering the regional hydrological regime, thereby increasing the complexity of the relationship between hydrological processes and riparian vegetation dynamics [27]. Before and after the operation of the Cuijiaying dam, the contribution of the climate to the NDVI of riparian vegetation showed little difference. However, after its operation, the contributions of precipitation and temperature to the vegetation NDVI were higher and lower, respectively, compared to before the operation. Before the second heightening of the Danjiangkou dam, the climate showed a greater contribution, whereas after the second heightening, the contributions of the climate and hydrology tended to balance (Table 9).

## 3. Discussion

**Figure 9:**
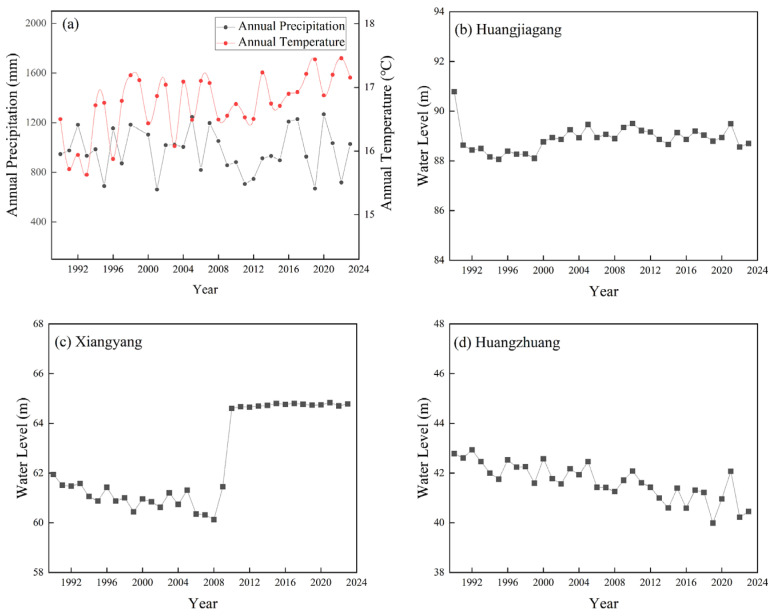
Changes in annual climate (precipitation and temperature) of the riparian (**a**) and water level at hydrological stations (**b**–**d**) of the middle reach of Hanjiang River from 1990 to 2023.

This research analyzed the spatiotemporal variations in riparian and typical floodplain vegetation under the influence of cascade hydropower development and their responses to the hydroclimate. In terms of the impact of cascade hydropower development on the growing season NDVI of riparian vegetation in the middle reach of Hanjiang River, we found that before the construction of the WFZ, there were more pixels with a decreasing trend in the growing season NDVI of riparian vegetation in the WFZ–CJY. However, the pixels with a decreased NDVI of riparian vegetation decreased after the completion of the WFZ, while most pixels showed an increasing trend in this region (Figure 4). The cause might be that the construction of the WFZ may control floods, reduce the impact of inundation on vegetation, and promote the growth of downstream vegetation [38]. Furthermore, this research found that the overall change in the annual average precipitation in the middle reach of Hanjiang River was not significant, while the average temperature generally showed an upward trend (Figure 9a). Given that a significant positive correlation between the riparian vegetation NDVI and annual temperature was found in this study (Table 2), and that the results also showed that the temperature was the main contributor to the changes in the NDVI after the WFZ and the first elevation of the DJK (Table 9), we suggest that dams may alter the regional hydrological regime, and this alteration in hydrological conditions may, in turn, have affected the relative contribution of temperature to vegetation dynamics. Whether this mechanism provides a plausible explanation for the observed increase in the vegetation NDVI after the operation of the WFZ and the first elevation of the DJK remains an open question worthy of further investigation.

This study found that the area of most floodplains was negatively correlated with the water level, which is similar to others’ research [22]. However, the area of P4 was significantly (*p* < 0.01) positively correlated with the water level. From 1990 to 2023, the area of P4 decreased, while the water level also decreased. It might be because the cascade hydropower development may trap sediment and intercept the downstream transportation of sediment to some degree [21]. On the one hand, cascade hydropower development helps control floods, but it may lead to a decrease in regional water levels and a slowdown in flow velocity [38], which are not conducive to the accumulation and expansion of P4. On the other hand, due to the reduction in sediment downstream, the sediment-starved flows commonly erode the P4 [39], resulting in decreasing the area of P4. We found that the mid-channel bars (M1, M2) have maintained a decreasing trend after the cascade hydropower development (Figure 6). We further calculated the variations in water level of hydrological stations corresponding to the typical floodplains. The water levels of hydrological stations (Huangjiagang and Xiangyang) corresponding to M1 and M2 increased after the cascade hydropower development (2013) (Figure 9b–d), and the water level at Xiangyang even experienced an abrupt change (in 2014) after the cascade development (Figure 8). Considering that the area of mid-channel bars was negatively correlated with the water level in this region (Table 4), our results suggest that dam regulation may result in a temporary increase in the water level (Figure 8), which likely causes inundations of mid-channel bars and results in area reductions in M1 and M2.

**Figure 10:**
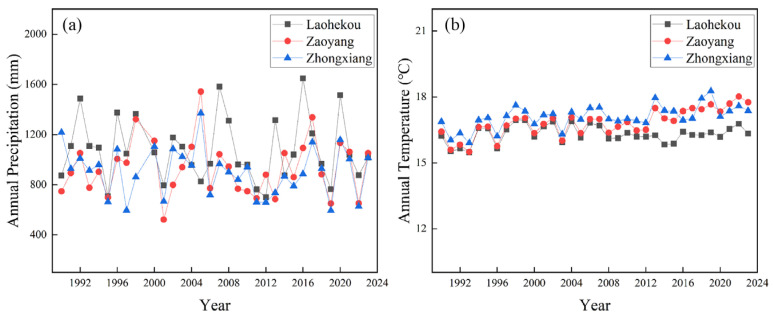
The variation trends in annual precipitation (**a**) and temperature (**b**) of different meteorological stations from 1990 to 2023.

To further analyze the influence of the climate on the vegetation NDVI, this study further analyzed the variation in annual precipitation and temperature from the meteorological stations corresponding to typical point and mid-channel bars in 1990–2023. It was found that the precipitation fluctuated, while the annual temperature generally showed an upward trend (Figure 10). We found that the typical point and mid-channel bars are generally positively correlated with temperatures in this study (Table 7 and Table 8), and the climate is the major contributor to the changes in the vegetation NDVI after the construction of the dam (Table 9). Our results suggest that increased temperatures potentially explain the increase in the vegetation NDVI of typical point and mid-channel bars to some extent (Figure 7).

This paper selects the Landsat data with higher resolution to synthesize the NDVI for processing. The NDVI can effectively represent vegetation vigor and its spatial distribution features [8,9], which is conducive to exploring the influence of the hydroclimate on the vegetation growth of riparian and typical floodplains. In order to better understand regional characteristics, it is necessary to further integrate multi-source data in future studies. In addition, the maximum value composite (MVC) method was utilized to reduce the influence of the atmosphere, clouds, etc. We should also realize that remote sensing data can still be affected by the other factors, thereby influencing the research results. To enhance the research accuracy, it is necessary to further integrate more optimized methods in future studies. In this study, the growing season was defined using an empirical approach informed by previous studies, which enables consistent long-term historical analysis [9,10]. Nevertheless, more comprehensive methods for dynamically identifying the growing season duration are important and should be further explored in future research. This study defines the riparian zone as a 5 km buffer from the main river channel to better reflect the variation characteristics of the vegetation NDVI under the influence of cascade hydropower development. However, considering the specificity of the region, this riparian zone may be insufficient to fully exclude areas that are not directly governed by water levels (such as upland vegetation, agricultural areas, and urban greening). Therefore, it is necessary to further explore the effects of water levels on different types of vegetation in the riparian zone to reduce the uncertainty in future research. The impacts of cascade hydropower development on vegetation are rather complex, but this study only considered the influence of a few factors of hydrology and climate. The solar radiation, inundation (frequency/duration, seasonal stage dynamics, or lagged effects), dam operations, sediment, the non-dam human activities (such as wetland park and bank reinforcement), and other environmental factors also affect the vegetation of riparian and typical floodplains. In order to better analyze the impact of hydrology and the climate on the vegetation of the basin under cascade hydropower development, the effect of more environmental factors on the vegetation of riparian and typical floodplains needs to be taken into consideration in future research.

## 4. Materials and Methods

### 4.1. Study Region

**Figure 11:**
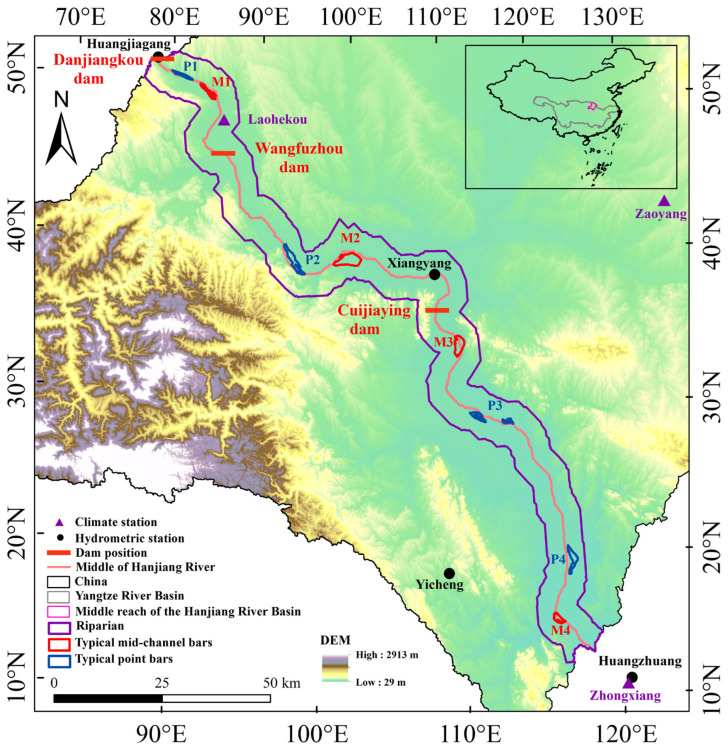
Study region (M means mid-channel bar and P means point bar, e.g., M1 means mid-channel bar 1).

The Hanjiang River, the largest tributary of the Yangtze River, serves as a vital water source for the Middle Route of the South-to-North Water Diversion Project and holds significant ecological and economic value [25,26] (Figure 11). The Hanjiang River originates in Ningqiang, Shaanxi Province, and flows into the Yangtze River in Wuhan, with a total length of 1577 km [40]. The upstream is the river’s origin to Danjiangkou, the middle is Danjiangkou to Zhongxiang, and the downstream is Zhongxiang to Wuhan [40]. The upstream has typical mountain ecosystems with narrow river valleys. The middle and downstream are mainly hilly and plain. The river valleys are relatively wide in the middle and downstream, but the downstream is widely distributed with artificial dams [29]. Due to the unique topography and the influence of cascade hydropower development, the middle reach of Hanjiang River is widely distributed with floodplains, such as point and mid-channel bars, which affect the regional ecological environment observably. This study selects the middle reach of Hanjiang River as the study area, and the total length of the middle reach of Hanjiang River is approximately 270 km. The basin has a subtropical monsoon climate with four distinct seasons [41,42]. The cascade hydropower development is widely distributed in the middle reach of Hanjiang River, including many important hydropower stations such as Danjiangkou dam (completed in 1973; first elevated in 2005; second elevated in 2013), Wangfuzhou dam (completed in 2003), Cuijiaying dam (completed in 2010), Xinji dam, Yakou dam, and so on [29]. In addition, the Yangtze-Hanjiang Water Diversion Project, as the subsequent water source project of the Middle Route of the South-to-North Water Diversion Project, also has significant value in alleviating the contradiction between regional water supply and demand and ensuring national water security [43,44]. With the development of cascade hydropower, the South-to-North Water Diversion Project, and the Yangtze-Hanjiang Water Diversion Project, the hydrological and ecological conditions in this basin have undergone varying degrees of changes.

### 4.2. Data

#### 4.2.1. Landsat Data

The 1990–2023 Landsat data (Landsat 5, Landsat 7, and Landsat 8) used in this research are all derived from the Google Earth Engine (GEE) platform. The temporal and spatial resolutions of the remote sensing data are 16 days and 30 m. This study defines the growing season as the research period to extract annual NDVI data.

#### 4.2.2. Hydrological and Meteorological Data

The monthly climate data (precipitation and temperature) during 1990–2023 are sourced from the China Meteorological Data Service Center (CMDC). The measured water level data of hydrological stations from 1990 to 2023 are sourced from the Hydrology and Water Resources Bureau of Hubei Province.

### 4.3. Methods

**Figure 12:**
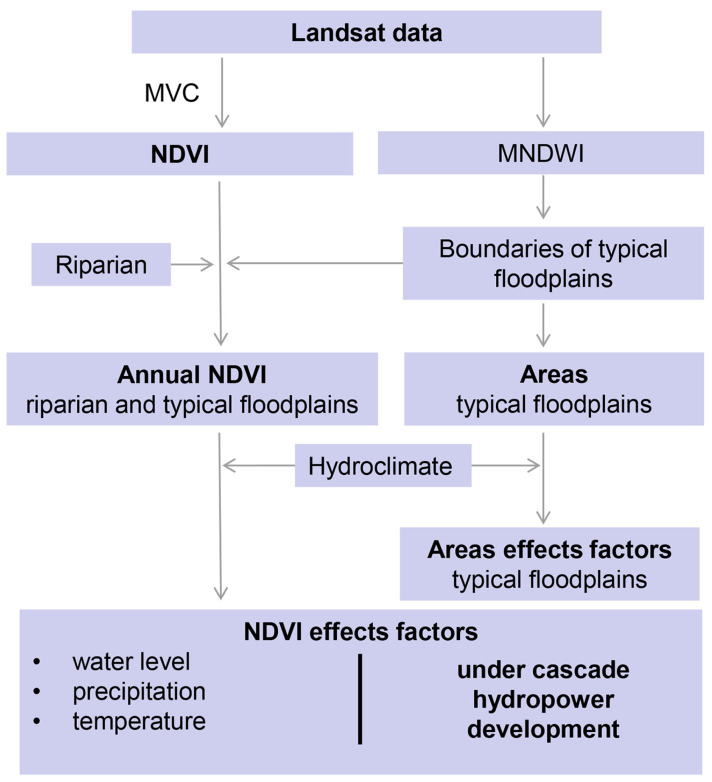
Flowchart of the analytical methods.

The sequence of methodologies is depicted in Figure 12, and we select the NDVI of vegetation during the growing season (April–October) for research to better understand the dynamic changes in vegetation [45,46], and the main vegetation types of this region are subtropical evergreen broad-leaved forest and wetland vegetation [45].

#### 4.3.1. Extraction of Riparian

Based on the existing research [17], this study uses ArcGIS (version 10.7) to generate a buffer zone within 5 km of the main river channel as the riparian zone, and takes the riparian zone as the study area to extract data.

#### 4.3.2. The Extraction of Point and Mid-Channel Bars

As floodplains respond to hydrological and climatic changes susceptibly, and considering the impact of the cascade hydropower development in the middle reach of Hanjiang River, this paper selects typical floodplains (point and mid-channel bars) in different river sections for analysis. The mid-channel bar 1 (M1) and point bar 1 (P1) belong to the Danjiangkou dam (DJK)–Wangfuzhou dam (WFZ). The mid-channel bar 2 (M2) and point bar 2 (P2) belong to the Wangfuzhou dam (WFZ)–Cuijiaying dam (CJY). The mid-channel bar 3 (M3) and point bar 3 (P3) belong to Cuijiaying dam (CJY)–Zhongxiang (ZX). The mid-channel bar 4 (M4) and point bar 4 (P4) are near Zhongxiang (ZX). In order to better clarify how cascade hydropower development impacts the growing season NDVI of typical point and mid-channel bars vegetation in the middle reach of Hanjiang River under the influence of the hydroclimate, this paper selects the data from the corresponding hydrological and meteorological stations that are closest to the typical point and mid-channel bars to analyze how the hydroclimate affects the vegetation NDVI.

Based on the riparian zones, for better extracting the distribution of typical point and mid-channel bars, GEE was used to generate the MNDWI during the dry season (November to March) in this region in each year, and ArcGIS was used to perform binary classification (the pixels of water bodies and land were assigned a value of 1 and 0) of the MNDWI index in each year. By combining visual interpretation, extracting the boundaries of typical point and mid-channel bars from each year, this paper superimposes the typical point and mid-channel bars boundary analysis morphological changes at different periods before and after the dam construction and calculates their areas.

$$MNDWI=\frac{\rho_{g}-\rho_{m}}{\rho_{g}+\rho_{m}}$$

In the formula, $\rho_{g}$ and $\rho_{m}$ represent the surface reflectance of the green band and the SWIR band, respectively.

#### 4.3.3. NDVI Calculation

Based on the Google Earth Engine platform, Landsat 5, 7, and 8 data were processed in a unified framework, including radiometric scaling using official scale factors and cloud/cloud-shadow masking based on the QA_PIXEL band. Consistent red and near-infrared band definitions were applied across sensors to ensure the consistency of the NDVI. For better eliminating the impacts of cloud cover, atmosphere, and so on, the maximum value composite (MVC) method [47] was used to obtain the annual NDVI of the growing season in study region. Based on the existing research [48], we extracted the pixels with NDVI > 0.2 as the vegetation distribution in this paper.

$$NDVI=\frac{\rho_{n}-\rho_{r}}{\rho_{n}+\rho_{r}}$$

In the formula, $\rho_{n}$ and $\rho_{r}$ are the surface reflectance of the green band of the NIR band and red band.

#### 4.3.4. Spatiotemporal Correlation Analysis

The Pearson correlation analysis method was used to analyze the relationship between the NDVI from the growing season and hydroclimate factors (*p* < 0.05 means significant correlation).

$$R_{xy}=\frac{\sum_{i=1}^{n}\left(x_{i}-\bar{x}\right)\left(y_{i}-\bar{y}\right)}{\sqrt{\sum_{i=1}^{n}{\left(x_{i}-\bar{x}\right)}^{2}}\sqrt{\sum_{i=1}^{n}{\left(y_{i}-\bar{y}\right)}^{2}}}$$

*R_xy_* refers to the correlation coefficient, *n* represents the length of the study period, *x_i_* references the average value of the hydrological or meteorological factor in year *i*, $\bar{x\;}$ refers to the multi-year average of the hydrological or meteorological factor during the study period, *y_i_* is the area value of typical floodplains or vegetation NDVI value in the riparian and typical floodplains in year *i*, and $\bar{y\;}$ is the multi-year average of the area value or vegetation NDVI value.

The Mann–Kendall (MK) test and simple linear regression were used to calculate the variation trend of hydroclimate factors and the spatial variation trend in the NDVI based on pixels in the research period.

$$\theta_{\mathrm{slope}}=\frac{\left(n\times \sum_{i=1}^{n}i\times B_{i}\right)\left(\sum_{i=1}^{n}i\times \sum_{i=1}^{n}B_{i}\right)}{n\times \sum_{i=1}^{n}i^{2}-{\left(\sum_{i=1}^{n}i\right)}^{2}}$$

*B_i_* represents the hydrological (climatic, vegetation NDVI) value of the year *i*, *i* refers to the serial number of the year, *n* represents the length of the research period, and *θ*_slope_ represents the trend slope of hydrological (climatic, vegetation NDVI) values. For each factor, *θ*_slope_ < 0 indicates a downward trend, *θ*_slope_ = 0 is no change, and *θ*_slope_ > 0 is an increase in the factor.

#### 4.3.5. The Effects of Hydroclimate Under the Cascade Hydropower Development

The Mann–Kendall trend test (MK test) was employed to detect trends and identify abrupt changes in the hydrology time series [25,29]. In this study, R (version 4.5.1) was used to implement the MK test to examine abrupt changes in water level and climatic time series, while minimizing the influence of outliers. Furthermore, we employed segmented multiple linear regression to further examine the relative importance of hydroclimate factors on the NDVI of riparian vegetation before and after the cascade hydropower development.

## 5. Conclusions

This research analyzed the spatiotemporal variations in riparian and typical floodplain vegetation under the influence of cascade hydropower development and further explored their key driving factors. This study found the following:

(1) The Normalized Difference Vegetation Index (NDVI) of the riparian vegetation in the middle reach of Hanjiang River showed an extremely significant (*p* < 0.01) increasing trend during the growing season from 1990 to 2023. The NDVI of the riparian vegetation and typical floodplains (point and mid-channel bars) all increased after the construction of the Wangfuzhou dam (2003), the first elevation increase in the Danjiangkou dam (2005), the construction of the Cuijiaying dam (2010), and the second elevation increase in the Danjiangkou dam (2013).

(2) The increase in the annual water level may explain the reducing areas of most point and mid-channel bars (except point bar 4), while the decreased annual water level may explain the reduction in point bar 4.

(3) The development of cascade hydropower may have caused the abrupt change in the water level that occurred at Xiangyang in 2014, and it may explain the different contributions of the climate and hydrology to the vegetation at different periods to a certain extent. This study found that the climate is the major contributor to the changes in the vegetation NDVI after the construction of the dams.

(4) Increasing temperature, especially increasing in March, can promote the vegetation growth of riparian and typical floodplains (point and mid-channel bars) during the growing season.

This study explored the key driving factors of variations in riparian and typical floodplain vegetation under cascade hydropower development in the middle reach of Hanjiang River, and provides a critical scientific basis for the protection and management of regional vegetation and riverine wetlands. It also implies that the area and shape of typical floodplains may affect vegetation growth, and this element should be considered in future research.

## Figures and Tables

**Table 1:** The average growing season NDVI of riparian vegetation in the middle reach of Hanjiang River before and after the operation or elevation of hydropower stations.

WFZ		DJK1		CJY		DJK2	
Before	After	Before	After	Before	After	Before	After
0.609	0.673	0.613	0.676	0.620	0.689	0.620	0.693

**Table 2:** Correlation analysis of growing season NDVI with climate and hydrological factors in the riparian zone (* and ** mean *p* < 0.05 and <0.01).

	Precipitation	Temperature	Water Level
Annual	0.153	0.433 *	0.023
January	0.208	0.146	−0.103
February	−0.137	−0.106	−0.077
March	−0.064	0.574 **	−0.056
April	0.248	0.065	−0.230
May	0.044	0.128	0.328
June	0.156	0.114	0.332
July	−0.035	0.138	0.208
August	−0.106	0.302	0.021
September	0.194	0.259	0.132
October	0.054	0.085	0.128
November	0.072	0.038	−0.266
December	−0.310	−0.105	−0.312

**Table 3:** The hydrological and meteorological stations correspond to typical point and mid-channel bars respectively (HS and MS mean hydrological station and meteorological station, DJK–WFZ means Danjiangkou dam–Wangfuzhou dam, WFZ–CJY means Wangfuzhou dam–Cuijiaying dam, CJY–ZX means Cuijiaying dam–Zhongxiang, Near ZX means near Zhongxiang).

	DJK–WFZ		WFZ–CJY		CJY–ZX		Near ZX	
	M1	P1	M2	P2	M3	P3	M4	P4
HS	Huangjiagang		Xiangyang		Xiangyang		Huangzhuang	
MS	Laohekou		Zaoyang		Zaoyang		Zhongxiang	

**Table 4:** The correlations between the areas of typical mid-channel bars and annual precipitation and water level (** mean *p* < 0.01).

	M1	M2	M3	M4
Precipitation	0.108	−0.188	−0.206	−0.176
Water level	−0.317	−0.731 **	−0.851 **	−0.639 **

**Table 5:** The correlations between the areas of typical point bars and annual precipitation and water level (* and ** mean *p* < 0.05 and <0.01).

	P1	P2	P3	P4
Precipitation	0.070	−0.225	−0.297	−0.013
Water level	−0.361 *	−0.434 *	−0.271	0.543 **

**Table 6:** The average NDVI of typical mid-channel and point bars vegetation before and after the hydropower station development.

	WFZ		DJK 1		CJY		DJK 2	
	Before	After	Before	After	Before	After	Before	After
M1	0.398	0.607	0.398	0.617	0.443	0.646	0.449	0.655
P1	0.470	0.644	0.462	0.646	0.497	0.678	0.500	0.690
M2	0.610	0.698	0.618	0.703	0.627	0.726	0.628	0.730
P2	0.505	0.633	0.509	0.636	0.526	0.666	0.534	0.660
M3	0.363	0.535	0.363	0.549	0.385	0.582	0.393	0.593
P3	0.386	0.660	0.404	0.674	0.461	0.673	0.478	0.680
M4	0.310	0.529	0.320	0.547	0.348	0.587	0.364	0.603
P4	0.415	0.678	0.445	0.690	0.487	0.709	0.499	0.729

**Table 7:** The correlations between climate and NDVI of mid-channel bars vegetation during the growing season (P and T mean precipitation and temperature, * and ** mean *p* < 0.05 and <0.01).

	M1		M2		M3		M4	
	P	T	P	T	P	T	P	T
Annual	0.039	−0.063	−0.076	0.641 **	0.057	0.626 **	−0.238	0.497 **
January	0.038	−0.104	−0.007	0.257	0.250	0.136	0.254	0.152
February	−0.188	−0.159	−0.127	0.076	−0.030	0.008	−0.356	0.012
March	−0.173	0.511 **	−0.148	0.645 **	0.156	0.535 **	0.205	0.553 **
April	0.040	−0.018	0.050	0.204	0.177	0.288	0.181	0.108
May	0.166	−0.038	−0.205	0.388 *	−0.062	0.251	−0.117	0.012
June	−0.007	0.008	0.124	0.369 *	−0.077	0.318	0.375	0.134
July	0.147	−0.196	−0.065	0.292	−0.022	0.249	−0.114	−0.014
August	−0.117	−0.028	−0.291	0.505 **	−0.056	0.498 **	−0.151	0.266
September	−0.058	−0.103	0.073	0.403 *	0.224	0.375 *	0.084	0.292
October	0.008	0.036	0.246	0.174	0.031	0.189	−0.125	0.121
November	−0.041	0.191	0.044	0.237	0.121	0.169	−0.075	0.146
December	−0.303	−0.264	−0.028	0.027	−0.316	0.126	−0.369	0.149

**Table 8:** The correlations between climate and NDVI of point bars vegetation during the growing season (P and T mean precipitation and temperature, * and ** mean *p* < 0.05 and <0.01).

	P1		P2		P3		P4	
	P	T	P	T	P	T	P	T
Annual	0.072	−0.009	−0.151	0.639 **	0.008	0.673 **	−0.206	0.508 **
January	−0.080	−0.038	0.046	0.127	0.299	0.013	0.206	0.168
February	−0.380 *	−0.137	−0.152	0.035	0.018	0.003	−0.247	0.008
March	−0.042	0.398 *	−0.182	0.622 **	−0.301	0.593 **	0.021	0.505 **
April	0.078	−0.053	−0.014	0.247	0.330	0.426 *	0.246	0.244
May	−0.035	0.003	−0.263	0.406 *	0.068	0.470 *	0.059	0.111
June	0.002	−0.103	0.084	0.417 *	−0.022	0.308	0.184	0.083
July	0.137	−0.148	−0.048	0.284	0.002	0.188	−0.118	0.070
August	−0.070	0.144	−0.261	0.419 *	−0.400 *	0.462 *	−0.220	0.220
September	−0.039	−0.052	0.034	0.346	0.227	0.167	0.113	0.198
October	0.231	−0.143	0.178	0.086	0.043	0.373	−0.045	0.158
November	0.038	0.079	0.024	0.223	0.218	0.014	0.013	0.106
December	−0.307	−0.078	0.000	0.069	−0.255	0.220	−0.323	0.121

**Table 9:** Relative importance of hydroclimate factors on riparian vegetation NDVI before and after the cascade hydropower development.

	WFZ		DJK1		CJY		DJK2	
	Before	After	Before	After	Before	After	Before	After
Precipitation	6.82%	41.78%	1.62%	47.69%	28.57%	53.25%	32.98%	41.81%
Temperature	16.78%	54.72%	33.08%	51.92%	56.90%	38.88%	56.81%	9.57%
Water level	76.41%	3.50%	65.29%	0.39%	14.53%	7.87%	10.21%	48.63%

## Data Availability

The raw data supporting the conclusions of this article will be made available by the authors on request.
